# Supplementary figures and images for: Amyloid-Beta Peptides Trigger Aggregation of Alpha-Synuclein In Vitro
Source: Molecules. 2020 Jan 29;25(3):580. doi: 10.3390/molecules25030580 (PMC7037551; doi:10.3390/molecules25030580)

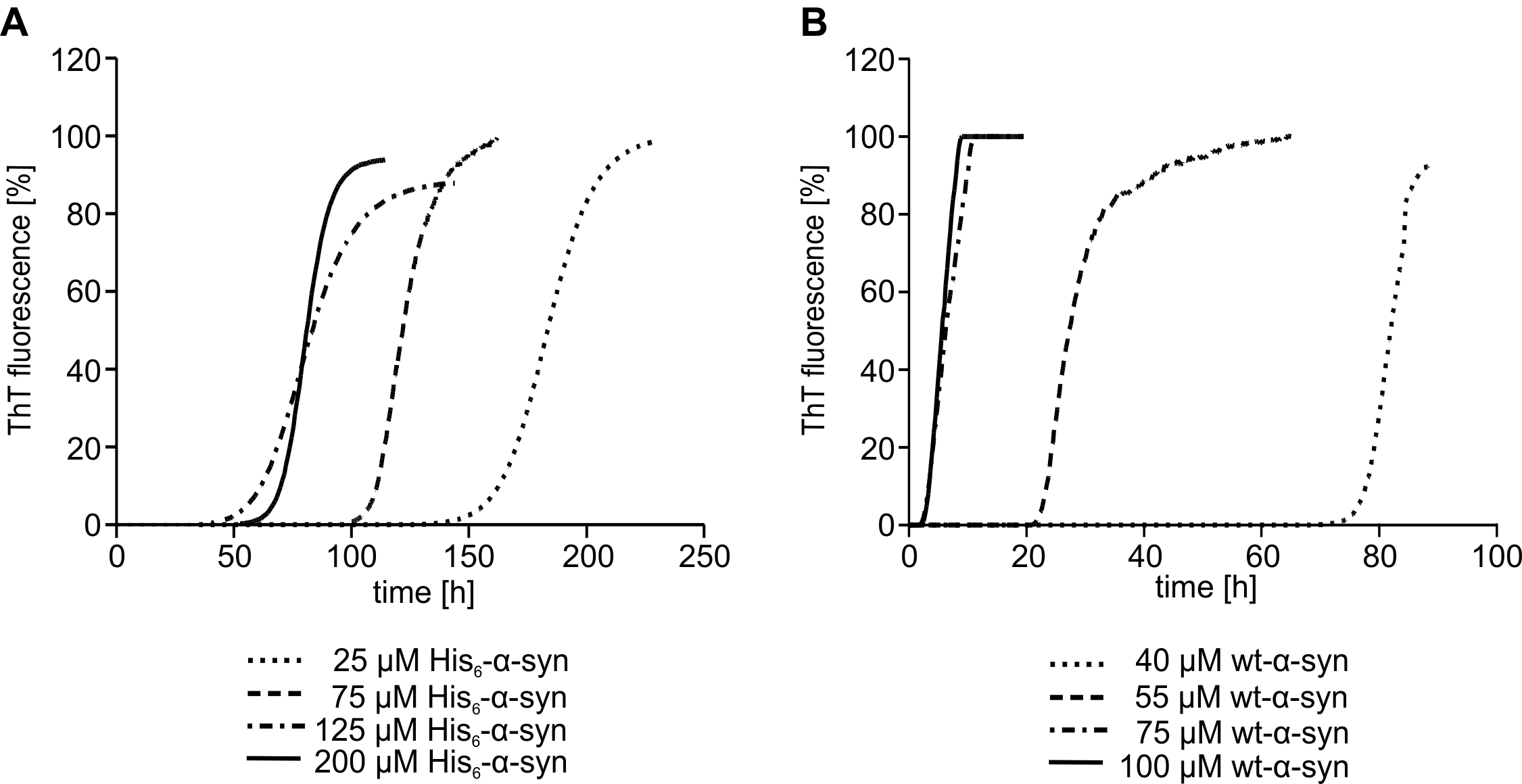

Supplement: Supplementary file 1 [file molecules-25-00580-s001.zip › Suppl Fig/Suppl. Figure 1.tif]

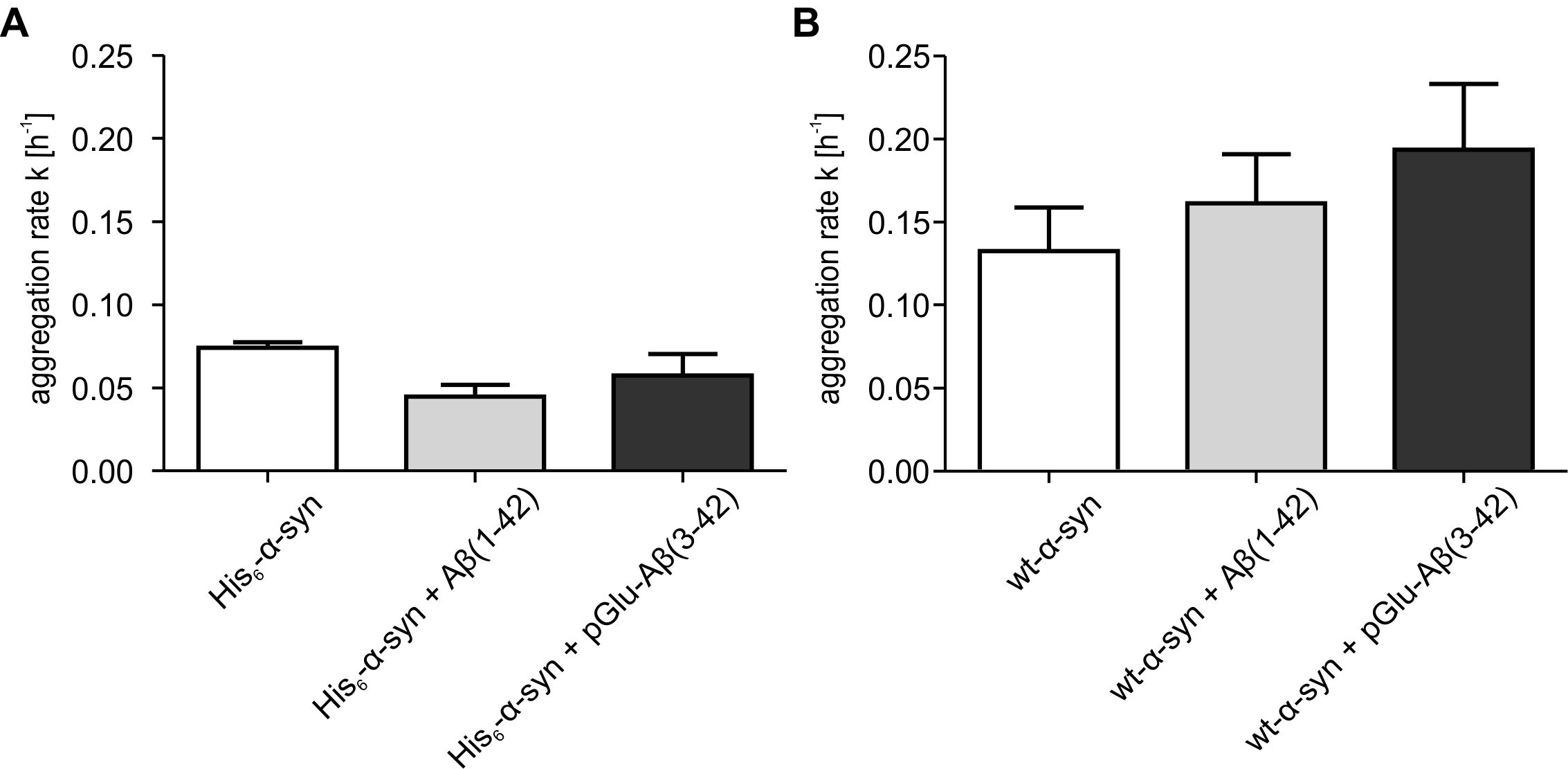

Supplement: Supplementary file 1 [file molecules-25-00580-s001.zip › Suppl Fig/Suppl. Figure 2.tif]
